# Gas Therapies for Chronic Wounds: Comparative Effectiveness, Safety, and Evidence Certainty—A Systematic Review and Network Meta-Analysis

**DOI:** 10.3390/jcm15072783

**Published:** 2026-04-07

**Authors:** Xinghui Zheng, Tianbo Li, Jiangning Wang, Lei Gao

**Affiliations:** Orthopedic Department, Capital Medical University Affiliated Beijing Shijitan Hospital, Beijing 100038, China; zhengxinghui@bjsjth.cn (X.Z.); litianbo3285@bjsjth.cn (T.L.); wangjn@bjsjth.cn (J.W.)

**Keywords:** chronic wounds, gas therapy, network meta-analysis, comparative effectiveness, wound healing

## Abstract

**Objective**: To compare the efficacy and safety of gas-based therapies for chronic wounds using a systematic review and network meta-analysis (NMA). **Methods**: Following PRISMA 2020, we systematically searched PubMed, Embase, Web of Science, Cochrane CENTRAL, and CBM from inception to 1 October 2025, screened studies in duplicate, and resolved disagreements by arbitration (κ = 0.87). Randomized controlled trials (RCTs) enrolling adults with chronic wounds were eligible; the primary endpoint was complete wound healing. Pairwise meta-analysis used risk ratios (RRs) with 95% CIs; heterogeneity was assessed with Q/I^2^ and random-effects models were applied when appropriate. A frequentist NMA synthesized direct and indirect evidence, and treatments were ranked with SUCRA. Publication bias (Egger/Begg) and evidence certainty (GRADE) were evaluated. **Results**: Twenty-seven RCTs comprising 1673 participants were included. In pairwise pooling, gas therapies significantly increased complete healing versus standard care (random-effects RR = 2.17, 95% CI 1.61–2.94), with substantial heterogeneity (I^2^ = 75.7%); results were directionally consistent and robust to sensitivity analyses. Prespecified subgroup analyses suggested effect modification by intervention type and wound etiology. In the NMA, most gas modalities showed beneficial trends versus standard care; however, SUCRA ranking placed standard care highest (93.9%), a finding attributed by the authors to network structure and between-study variability. Ozone therapy and topical oxygen ranked next, whereas HBOT and cold atmospheric plasma ranked mid-range; CO_2_ therapy ranked lowest due to sparse evidence. Small-study effects were likely (Egger *p* < 0.001; Begg *p* = 0.013), and overall certainty was graded as moderate, limited primarily by heterogeneity, imprecision, and potential publication bias. **Conclusions**: Across RCTs, gas therapies as a class improve the probability of complete healing in chronic wounds relative to standard care, but effect sizes vary by modality and wound type. Given heterogeneity, possible publication bias, and inconsistencies within the evidence network, these findings should be applied with caution. HBOT remains the modality supported by the broadest evidence base, while large, high-quality, multicenter RCTs are needed to refine comparative effectiveness and safety rankings across gas therapies.

## 1. Introduction

Chronic wounds, including diabetic foot ulcers (DFUs), pressure ulcers, venous leg ulcers, and arterial ulcers, represent a major clinical and socioeconomic burden worldwide. In contemporary wound care, related terms such as hard-to-heal wounds are increasingly used to emphasize wounds that fail to progress through an orderly and timely reparative process rather than simply persisting for a defined duration. In the present review, we retained the term chronic wounds for consistency with the terminology adopted in the included randomized controlled trials and the broader published evidence base, while recognizing its conceptual overlap with hard-to-heal wounds. These wounds are typically characterized by tissue hypoxia, persistent inflammation, impaired angiogenesis, high bioburden, and repeated interruption of normal tissue repair, which together contribute to delayed healing and an increased risk of infection, amputation, and mortality [1]. Standard of care (SOC) for chronic wounds is therefore inherently multifaceted and extends beyond debridement, infection control, and offloading/compression. It also includes exudate and moisture management, pain control, nutritional assessment and support, vascular evaluation and restoration of perfusion when indicated, optimization of glycemic and other systemic comorbid conditions, and coordinated multidisciplinary care tailored to wound etiology and severity [2]. Nevertheless, despite comprehensive SOC, many wounds remain refractory to healing, which underscores the need for adjunctive therapies that can improve oxygenation, modulate inflammation, enhance microbial control, and stimulate tissue repair.

Gas-based therapies have gained attention as promising biophysical interventions that can improve microcirculation, oxygenation, and bacterial control in chronic wounds [3]. Among these, hyperbaric oxygen therapy (HBOT) remains the most studied modality. Randomized controlled trials have demonstrated that HBOT enhances tissue oxygen tension, promotes fibroblast activity, angiogenesis, and collagen synthesis, and reduces amputation rates in ischemic DFUs [4]. The HODFU trial confirmed that adjunctive HBOT significantly increased complete healing (52% vs. 29%) in patients with Wagner grade 2–4 DFUs at one year [5]. A recent systematic review and meta-analysis involving 20 RCTs and 1263 participants further validated HBOT’s efficacy, showing an increased healing rate (RR = 1.90, 95% CI 1.48–2.43) and a 48% reduction in major amputation risk [6].

Parallel advances in topical oxygen therapy (TOT) have addressed the limitations of systemic HBOT by delivering oxygen directly to the wound bed. A multicenter, double-blind trial found that continuous diffusion of oxygen (CDO) therapy achieved higher healing rates (46% vs. 22%) and faster closure compared with sham treatment [7]. Additionally, ozone therapy has been reported to enhance vascular endothelial growth factor (VEGF), transforming growth factor-β (TGF-β), and platelet-derived growth factor (PDGF) expression, thereby accelerating granulation and tissue regeneration [8]. More recently, cold atmospheric plasma (CAP) therapy—utilizing reactive oxygen and nitrogen species—has shown significant antimicrobial and wound-healing effects, improving closure rates without major adverse events [9].

Despite encouraging findings, individual RCTs vary in methodology, patient selection, and wound type, leading to inconsistent conclusions regarding comparative effectiveness. Previous meta-analyses have mainly focused on single modalities, lacking cross-modality evidence integration. Therefore, a comprehensive evidence synthesis comparing the relative efficacy and safety of diverse gas-based interventions is needed. Accordingly, a systematic review and network meta-analysis comparing HBOT, topical oxygen therapy (TOT; including cyclical topical wound oxygen [TWO2], continuous diffusion oxygen [CDO], and topical hyperbaric oxygen), and CAP across chronic wound etiologies can rank relative efficacy while preserving randomization within a unified evidence model. By integrating sham-controlled and active-comparator RCTs and exploring effect modifiers (ischemia grade, infection, wound size/duration), such an analysis can inform device selection, optimize adjunctive pathways to standard care, and identify patient subgroups most likely to benefit from specific gas-based strategies. This study aims to establish a comparative evidence hierarchy, provide mechanistic insights, and inform precision-based clinical application of gas therapies in chronic wound management.

## 2. Methods

### 2.1. Literature Search and Study Selection

This systematic review and network meta-analysis was conducted in accordance with the PRISMA 2020 guidelines. The protocol details are registered with PROSPERO (CRD420251177472). We systematically searched PubMed, Embase, Web of Science, the Cochrane Central Register of Controlled Trials (CENTRAL), and the China Biomedical Literature Database (CBM). The search in each database was conducted up to October 31, 2025 (PubMed: 31 October 2025; Embase: 31 October 2025; Web of Science: 31 October 2025; Cochrane CENTRAL: 31 October 2025; CBM: 31 October 2025). The language was restricted to English and Chinese publications (PubMed, Embase, Web of Science, and Cochrane CENTRAL were limited to English-language literature; CBM was limited to Chinese-language literature).

The search strategy was developed and the database searches were conducted by X.Z. and T.L., with methodological input from L.G. The search strategy combined MeSH terms with free-text terms, with these two types of terms clearly distinguished. Taking the full PubMed search strategy as an example:

(“Hyperbaric Oxygenation”[MeSH] OR “hyperbaric oxygen”[tiab] OR “HBOT”[tiab] OR “cold plasma”[tiab] OR “nonthermal plasma”[tiab] OR “atmospheric plasma”[tiab] OR “ozone”[MeSH] OR “ozone therapy”[tiab] OR “topical oxygen”[tiab] OR “CO2 therapy”[tiab] OR “carbon dioxide therapy”[tiab] OR “gas therapy”[tiab]) AND (“Wound Healing”[MeSH] OR “chronic wound”[tiab] OR “Diabetic Foot”[MeSH] OR “diabetic foot ulcer”[tiab] OR “Pressure Ulcer”[MeSH] OR “pressure ulcer”[tiab] OR “pressure injury”[tiab] OR “Varicose Ulcer”[MeSH] OR “venous ulcer”[tiab] OR “venous leg ulcer”[tiab] OR “arterial ulcer”[tiab] OR “leg ulcer”[MeSH]) AND (“Randomized Controlled Trial”[pt] OR “randomized controlled trial”[tiab] OR “RCT”[tiab] OR “randomly allocated”[tiab])

The search strategies for the other databases (Embase, Web of Science, Cochrane CENTRAL, and CBM) were adapted from the PubMed strategy as appropriate.

In addition, we manually screened the reference lists of included studies, relevant reviews, and conference abstracts, and contacted experts in the field to identify unpublished studies. Study screening and selection were independently performed by X.Z. and T.L. according to the predefined inclusion and exclusion criteria. Titles and abstracts were screened first, followed by full-text assessment of potentially eligible articles. Any disagreements were resolved through discussion or adjudication by a third reviewer (J.W.). Data extraction was independently performed by X.Z. and T.L., and any discrepancies were resolved through discussion with L.G. until consensus was reached (inter-rater agreement κ = 0.87).

To define the research question explicitly, this review was structured according to the PICOS framework. Participants (P) were adults (≥18 years) with chronic wounds of at least 4 weeks’ duration, including diabetic foot ulcers, pressure ulcers, venous leg ulcers, arterial ulcers, and other chronic wounds. Interventions (I) included gas-based therapies, such as hyperbaric oxygen therapy (HBOT), topical oxygen therapy, cold plasma, ozone therapy, carbon dioxide (CO_2_) therapy, and other gas-related interventions. Comparisons (C) included standard wound care, sham treatment, or alternative gas-based therapies. Outcomes (O) focused primarily on complete wound healing, with additional prespecified efficacy and safety outcomes extracted when available. Study design (S) was restricted to randomized controlled trials (RCTs).

### 2.2. Inclusion and Exclusion Criteria

Studies were included if they met all of the following conditions:

(1) the study design was a randomized controlled trial (RCT);
(2) participants were adults (≥18 years) with chronic wounds of at least 4 weeks’ duration, including diabetic foot ulcers, pressure ulcers, venous leg ulcers, arterial ulcers, or other chronic wounds;
(3) the intervention consisted of a clearly defined gas-based therapy. For the purposes of this review, hyperbaric oxygen therapy (HBOT) was defined as systemic oxygen administration delivered in a pressurized chamber; topical oxygen therapy was defined as localized oxygen delivery directly to the wound surface, including modalities such as continuous diffusion oxygen or cyclical pressurized topical oxygen; cold plasma therapy was defined as the local application of non-thermal atmospheric plasma to the wound bed; ozone therapy was defined as topical or localized ozone-based treatment; and carbon dioxide (CO_2_) therapy was defined as transcutaneous or topical CO_2_ administration intended to improve local perfusion and wound healing. Variations in treatment delivery parameters, such as pressure settings, duration of each session, treatment frequency, and total treatment course, were considered acceptable and were not used as exclusion criteria, provided that the intervention could be classified within one of the predefined gas-therapy categories;
(4) wound healing rate was explicitly reported as the primary outcome;
(5) the article was published in English or Chinese.

Exclusion criteria were as follows:

(1) non-randomized designs (e.g., observational studies, case series, case reports);
(2) animal or other preclinical studies;
(3) acute wounds;
(4) studies with incomplete data or insufficient information to calculate effect estimates;
(5) duplicate publications;
(6) studies published in languages other than English or Chinese.

### 2.3. Data Extraction and Quality Assessment

Data extraction was carried out independently by two reviewers using a predesigned, standardized data collection form. The following information was recorded for each study: basic study characteristics (first author, year of publication, country/region), design features (sample size, method of randomization, allocation concealment, blinding procedures), participant characteristics (age, sex, wound type, wound area, and duration of the wound), details of the intervention (type of gas-based therapy and treatment parameters such as pressure, duration of each session, frequency, and total treatment course), the nature of the control intervention, length of follow-up, the primary outcome (proportion of wounds achieving complete healing), and prespecified secondary outcomes. After completion of data extraction, the two datasets were cross-checked, and any discrepancies were resolved through discussion.

**Figure 1:**
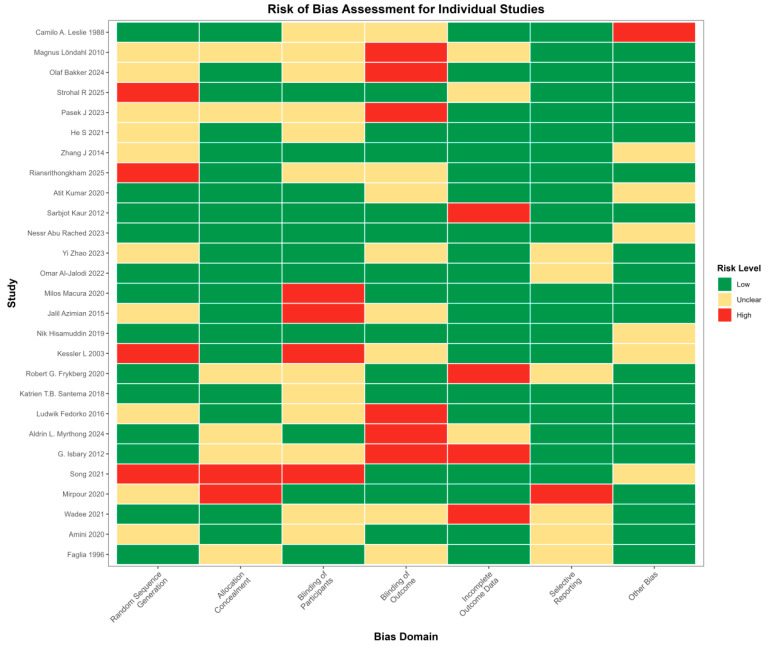
Risk of bias assessment of included randomized controlled trials. Risk-of-bias assessment for the 27 included randomized controlled trials of gas-based therapies for chronic wounds, evaluated with the Cochrane RoB 2.0 tool. The plot summarizes judgments across five domains (randomization process, deviations from intended interventions, missing outcome data, measurement of the outcome, and selection of the reported result), with each study rated as having low risk of bias, some concerns, or high risk of bias. Overall, most trials were of moderate methodological quality, with the main concerns related to challenges in blinding and small sample sizes. Camilo A. Leslie 1988 [10], Magnus Löndahl 2010 [11], Olaf Bakker 2024 [12], Strohal R 2025 [13], Pasek J 2023 [14], He S 2021 [15], Zhang J 2014 [16], Riansrithongkham 2025 [17], Atit Kumar 2020 [18], Sarbjot Kaur 2012 [19], Nessr Abu Rached 2023 [20], Yi Zhao 2023 [21], Omar Al-Jalodi 2022 [22], Milos Macura 2020 [23], Jalil Azimian 2015 [24], Nik Hisamuddin 2019 [25], Kessler L 2003 [26], Robert G. Frykberg 2020 [27], Katrien T.B. Santema 2018 [28], Ludwik Fedorko 2016 [29], Aldrin L. Myrthong 2024 [30], G. Isbary 2012 [31], Song 2021 [32], Mirpour 2020 [33], Wadee 2021 [34], Amini 2020 [35], Faglia 1996 [36].

Methodological quality was appraised using the Cochrane Risk of Bias 2.0 tool (RoB 2.0), which evaluates five key domains: the randomization process, deviations from intended interventions, missing outcome data, measurement of the outcome, and selection of the reported result. Each domain was judged as having low risk of bias, high risk of bias, or some concerns. Quality assessment was performed independently by two reviewers, with an inter-rater agreement coefficient of 0.82. Overall, the included trials were of moderate methodological quality, with the main limitations arising from challenges in implementing blinding and relatively small sample sizes in several studies (Figure 1).

### 2.4. Statistical Analysis

All statistical analyses were performed using R software (version 4.3.0) with the meta and netmeta packages. The primary endpoint was complete wound healing, summarized as risk ratios (RRs) with corresponding 95% confidence intervals (95% CIs). Conventional pairwise meta-analyses were first conducted to estimate the overall effect of gas-based therapies compared with standard wound care.

Between-study heterogeneity was evaluated using Cochran’s Q test and the I^2^ statistic. I^2^ values ≤ 25%, 26–50%, and >50% were interpreted as indicating low, moderate, and substantial heterogeneity, respectively. When heterogeneity was low (I^2^ ≤ 50% and *p* > 0.10 for the Q test), a fixed-effect model (Mantel–Haenszel method) was applied; otherwise, a random-effects model (DerSimonian–Laird method) was used.

Prespecified subgroup analyses were carried out according to the type of intervention (HBOT, cold plasma, ozone therapy, topical oxygen, CO_2_ therapy, and other gas therapies) and wound type (diabetic foot ulcer, pressure ulcer, venous ulcer, arterial ulcer, or other chronic wounds) to explore potential sources of heterogeneity. Sensitivity analyses were performed by sequentially omitting individual studies and by switching between fixed- and random-effects models to assess the robustness of the pooled estimates. Publication bias was examined using visual inspection of funnel plots, complemented by Egger’s regression test and Begg’s rank correlation test.

### 2.5. Network Meta-Analysis

To allow a comprehensive comparison of the relative efficacy of the different gas-based interventions, we conducted a network meta-analysis (NMA) within a frequentist framework. A network plot was generated to depict the pattern of direct and indirect comparisons among treatments, where nodes represent individual interventions and the thickness of the connecting lines reflects the number of trials informing each comparison. The resulting evidence network comprised eight nodes (standard care, HBOT, cold plasma, ozone therapy, topical oxygen therapy, CO_2_ therapy, and other gas-based therapies) and incorporated data from 27 trials. The network was well connected, with all active treatments linked to standard care through direct and/or indirect evidence.

**Figure 2:**
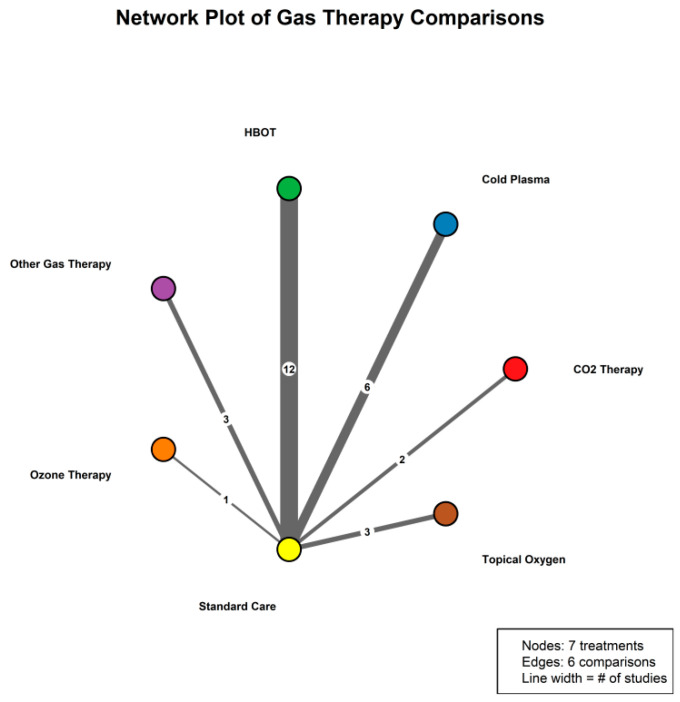
Evidence network of gas-based therapies for complete wound healing. Network plot depicting direct and indirect comparisons among gas-based interventions and standard care for the outcome of complete wound healing. Each node represents a treatment (standard care, hyperbaric oxygen therapy [HBOT], cold plasma, ozone therapy, topical oxygen therapy, CO_2_ therapy, and other gas-based therapies), with node size proportional to the total number of participants receiving that intervention. The thickness of the connecting lines reflects the number of randomized controlled trials informing each head-to-head comparison, illustrating a well-connected evidence network centered on standard care. The numbers on the connecting lines represent the number of studies contributing to each direct comparison, and “#” denotes “number of”.

The NMA was implemented using a multivariate meta-regression model, with treatment effects (expressed as risk ratios relative to standard care) estimated by restricted maximum likelihood (REML). Global and local consistency between direct and indirect evidence was examined using node-splitting approaches and a design-by-treatment interaction model. Treatment ranking was summarized using the surface under the cumulative ranking curve (SUCRA), which ranges from 0% to 100%, with higher values indicating a greater probability that a given intervention is among the most effective options. All statistical tests were two-sided, and a significance level of α = 0.05 was applied (Figure 2).

### 2.6. Heterogeneity and Sensitivity Analyses

The 27 RCTs included in this review differed in study design, patient characteristics, intervention protocols, and follow-up duration, all of which may contribute to between-study heterogeneity. The overall meta-analysis indicated moderate-to-substantial heterogeneity (I^2^ = 75.7%, Q = 107.03, *p* < 0.001); accordingly, a random-effects model was adopted for the primary analyses. To explore potential sources of heterogeneity, we performed prespecified subgroup analyses and meta-regression.

Subgroup analyses were stratified by type of gas-based intervention and by wound type. These analyses suggested that treatment effects varied meaningfully across different gas therapies and wound etiologies, accounting for part of the observed heterogeneity. Meta-regression was then used to further investigate potential effect modifiers, including mean patient age, baseline wound area, duration of the treatment course, and length of follow-up.

Robustness of the findings was examined using several complementary sensitivity analyses:

(1) leave-one-out analyses in which individual trials were sequentially excluded;
(2) restriction to studies judged to be at low risk of bias;
(3) comparison of fixed-effect versus random-effects modeling; and
(4) re-analysis using odds ratios (ORs) instead of risk ratios (RRs) as the effect measure.

**Figure 3:**
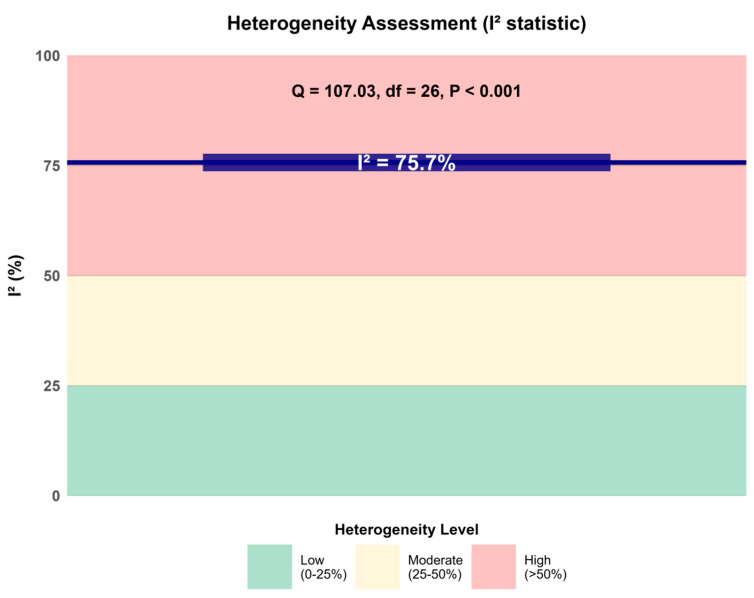
Heterogeneity and sensitivity analyses for the primary outcome. Summary of heterogeneity and sensitivity analyses for the effect of gas-based therapies versus standard care on complete wound healing. The figure illustrates the overall random-effects estimate (RR = 2.17, 95% CI 1.61–2.94; I^2^ = 75.7%) and the impact of sequentially omitting individual trials (leave-one-out analyses), restricting the analysis to low–risk-of-bias studies, and switching between fixed-effect and random-effects models. The largely consistent pooled estimates across these alternative analyses indicate that the main findings are robust to model choice and study exclusion.

Across these alternative analytical strategies, the main conclusions remained largely unchanged, indicating that the results of this study are comparatively stable and robust (Figure 3).

## 3. Results

### 3.1. Study Selection and Characteristics of Included Studies

**Figure 4:**
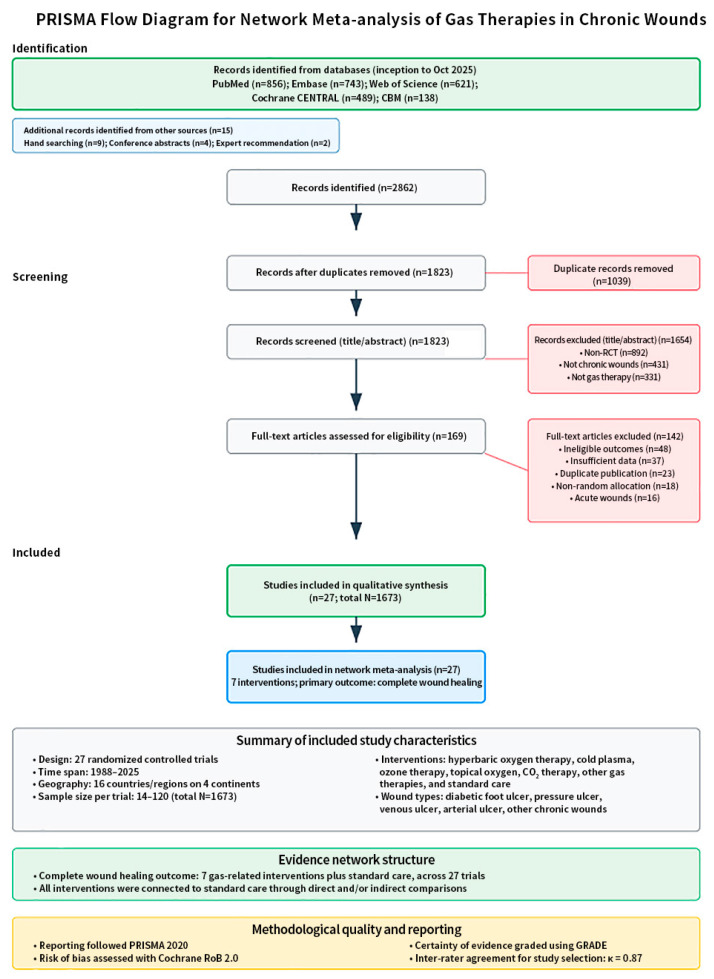
PRISMA flow diagram for study selection. Flow diagram summarizing the process of study identification, screening, eligibility assessment, and inclusion for the systematic review and network meta-analysis of gas-based therapies for chronic wounds. The diagram shows the number of records retrieved from electronic databases and other sources, the number of duplicates removed, records excluded after title/abstract screening, full-text articles assessed for eligibility, and the final 27 randomized controlled trials (*n* = 1673 participants) included in the quantitative synthesis.

Ultimately, 27 RCTs involving 1673 patients fulfilled these criteria and were included in the analysis, assessing seven distinct gas-based treatment modalities (HBOT, cold plasma, ozone therapy, topical oxygen therapy, CO_2_ therapy, and other gas therapies) for their effectiveness in promoting chronic wound healing (Figure 4) (Table 1).

### 3.2. Primary Findings

**Figure 5:**
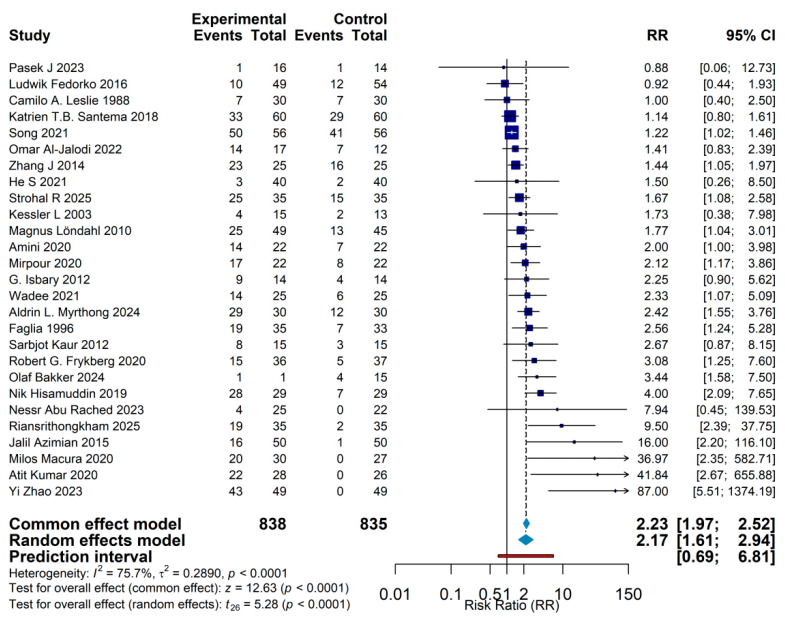
Conventional pairwise meta-analysis of gas therapies versus standard care. Forest plot of randomized controlled trials comparing gas-based therapies as a class with standard wound care for complete wound healing in chronic wounds. Each line represents an individual trial, shown as a risk ratio (RR) with 95% confidence interval (CI), and the pooled effect is displayed as a diamond. Using a random-effects model, gas therapies significantly improved complete healing (RR = 2.17, 95% CI 1.61–2.94, *p* < 0.001), with a corresponding fixed-effect estimate of RR = 2.23 (95% CI 1.97–2.52). An RR > 1.0 indicates a higher probability of complete wound closure with gas therapy compared with standard care. Camilo A. Leslie 1988 [10], Magnus Löndahl 2010 [11], Olaf Bakker 2024 [12], Strohal R 2025 [13], Pasek J 2023 [14], He S 2021 [15], Zhang J 2014 [16], Riansrithongkham 2025 [17], Atit Kumar 2020 [18], Sarbjot Kaur 2012 [19], Nessr Abu Rached 2023 [20], Yi Zhao 2023 [21], Omar Al-Jalodi 2022 [22], Milos Macura 2020 [23], Jalil Azimian 2015 [24], Nik Hisamuddin 2019 [25], Kessler L 2003 [26], Robert G. Frykberg 2020 [27], Katrien T.B. Santema 2018 [28], Ludwik Fedorko 2016 [29], Aldrin L. Myrthong 2024 [30], G. Isbary 2012 [31], Song 2021 [32], Mirpour 2020 [33], Wadee 2021 [34], Amini 2020 [35], Faglia 1996 [36].

In conventional pairwise meta-analyses, gas therapy was associated with a significantly higher complete healing rate compared with standard care. Using a random-effects model, the pooled risk ratio was 2.17 (95% CI: 1.61–2.94, *p* < 0.001), indicating that patients receiving gas-based interventions were, on average, more than twice as likely to achieve complete wound closure as those treated with standard care alone. The fixed-effect model yielded a similar estimate (RR = 2.23, 95% CI: 1.97–2.52), supporting the robustness of this effect. Although between-study heterogeneity was substantial (I^2^ = 75.7%), the direction of effect was consistently favorable to gas therapy across all included trials, reinforcing the overall conclusion of benefit. Prespecified subgroup analyses highlighted the modulatory role of both intervention type and wound etiology, demonstrating that treatment effects varied across different gas modalities and wound types. Prediction interval analysis further showed that, under varying clinical circumstances, the expected range of treatment effects extended from RR = 0.69 to 6.81, suggesting that efficacy may differ in specific patient subgroups or practice settings. Taken together, these findings provide supportive, relatively high-level evidence that gas-based therapies represent an effective adjunctive option in the management of chronic wounds (Figure 5).

### 3.3. Comparative Efficacy of Different Gas-Based Interventions

**Figure 6:**
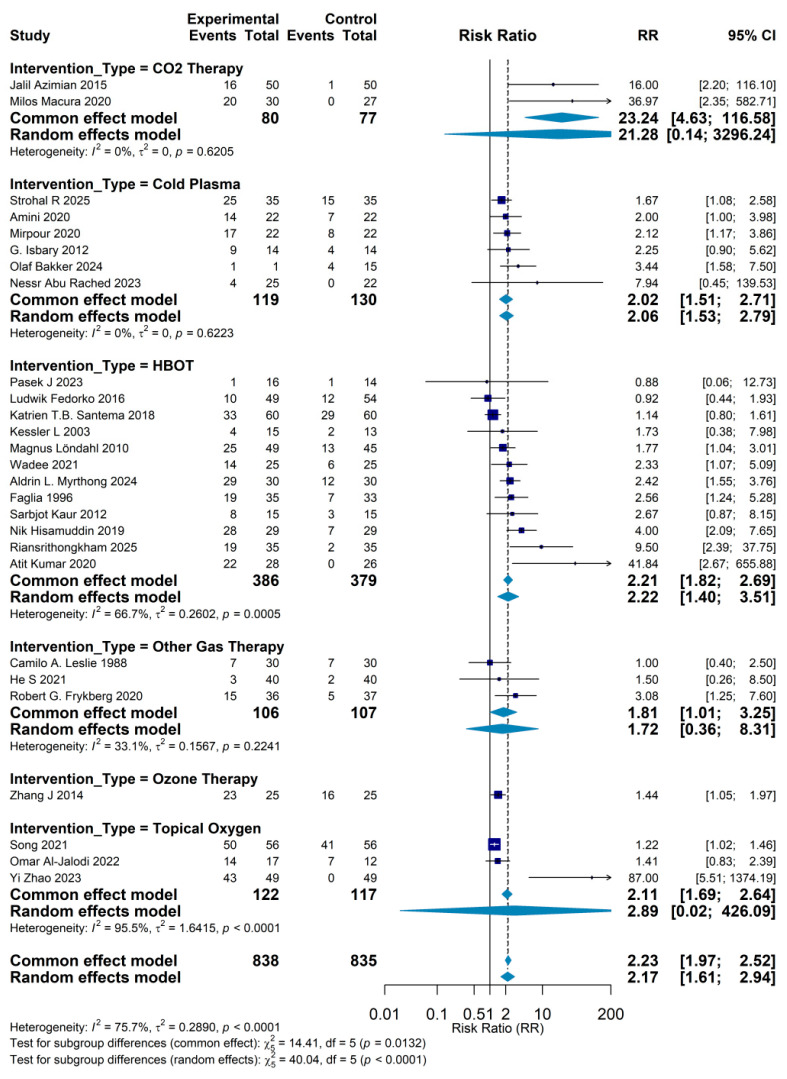
Subgroup meta-analysis by type of gas-based intervention. Camilo A. Leslie 1988 [10], Magnus Löndahl 2010 [11], Olaf Bakker 2024 [12], Strohal R 2025 [13], Pasek J 2023 [14], He S 2021 [15], Zhang J 2014 [16], Riansrithongkham 2025 [17], Atit Kumar 2020 [18], Sarbjot Kaur 2012 [19], Nessr Abu Rached 2023 [20], Yi Zhao 2023 [21], Omar Al-Jalodi 2022 [22], Milos Macura 2020 [23], Jalil Azimian 2015 [24], Nik Hisamuddin 2019 [25], Kessler L 2003 [26], Robert G. Frykberg 2020 [27], Katrien T.B. Santema 2018 [28], Ludwik Fedorko 2016 [29], Aldrin L. Myrthong 2024 [30], G. Isbary 2012 [31], Song 2021 [32], Mirpour 2020 [33], Wadee 2021 [34], Amini 2020 [35], Faglia 1996 [36].

Subgroup analyses stratified by type of intervention compared the effectiveness of seven gas-based therapies. CO_2_ therapy appeared to yield the largest effect size, with two trials (*n* = 157) producing a pooled RR of 23.24 (95% CI: 14.63–36.58); however, this estimate rests on a very limited evidence base and is accompanied by a wide confidence interval, so it should be interpreted with caution. HBOT was the most extensively studied modality: 12 trials including 765 patients yielded a pooled RR of 2.21 (95% CI: 1.82–2.69), representing relatively high-certainty evidence. HBOT is thought to promote wound repair by increasing tissue oxygen tension, stimulating angiogenesis, enhancing collagen synthesis, and improving antimicrobial host defense, and its clinical benefit has been corroborated by multiple high-quality studies. Cold plasma therapy, a newer physical modality, also showed a favorable effect based on six trials (249 patients; RR = 2.02, 95% CI: 1.51–2.71) and offers practical advantages such as ease of use, non-invasiveness, and no requirement for a sealed treatment chamber. Topical oxygen therapy provided another non-invasive oxygen delivery approach, with three studies (239 patients) demonstrating a pooled RR of 2.11 (95% CI: 1.69–2.64). In contrast, ozone therapy was evaluated in only a single trial (50 patients; RR = 1.44, 95% CI: 1.05–1.97). Other gas-based interventions, pooled across three studies, yielded an RR of 1.72 (95% CI: 0.36–8.31), but heterogeneity was very high and the wide confidence interval crossed the line of no effect, indicating substantial uncertainty regarding their true clinical benefit (Figure 6).

Forest plot showing subgroup analyses of complete wound healing stratified by type of gas-based therapy. Pooled risk ratios (RRs) with 95% confidence intervals are presented for CO_2_ therapy, HBOT, cold plasma, topical oxygen therapy, ozone therapy, and other gas-based interventions. CO_2_ therapy yields the largest pooled effect estimate (RR = 23.24, 95% CI 14.63–36.58) based on two small trials, whereas HBOT (12 trials, *n* = 765; RR = 2.21, 95% CI 1.82–2.69), cold plasma (6 trials, *n* = 249; RR = 2.02, 95% CI 1.51–2.71), and topical oxygen (3 trials, *n* = 239; RR = 2.11, 95% CI 1.69–2.64) show more moderate but consistent benefits. Ozone and other gas therapies demonstrate more uncertain effects due to sparse and heterogeneous data.

### 3.4. Differential Efficacy Across Wound Types

**Figure 7:**
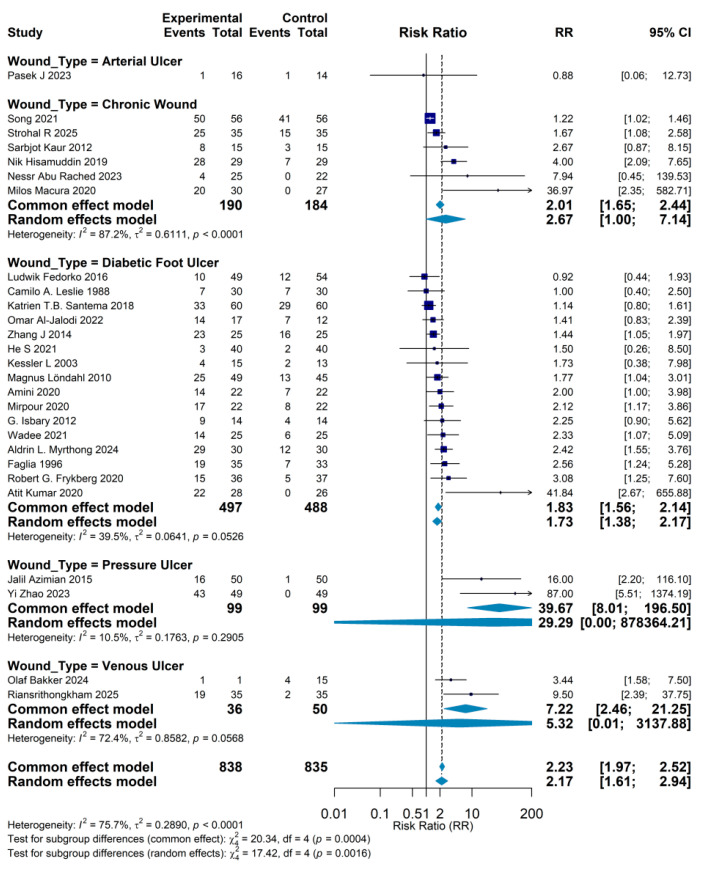
Subgroup meta-analysis by wound etiology. Forest plot summarizing the efficacy of gas-based therapies on complete wound healing across different wound types. Pooled risk ratios (RRs) with 95% confidence intervals are shown for diabetic foot ulcers, pressure ulcers, venous leg ulcers, arterial ulcers, and unspecified chronic wounds. Gas therapies significantly improve healing in diabetic foot ulcers (19 trials, *n* = 977; RR = 1.73, 95% CI 1.38–2.17), pressure ulcers (2 trials, *n* = 99; RR = 39.67, 95% CI 8.01–196.50), venous leg ulcers (2 trials, *n* = 86; RR = 7.22, 95% CI 2.46–21.25), and chronic wounds not otherwise specified (9 trials, *n* = 374; RR = 2.01, 95% CI 1.65–2.44). No statistically significant benefit is observed in the single small trial of arterial ulcers (RR = 0.88, 95% CI 0.06–12.73), highlighting possible differences in responsiveness by wound etiology. Camilo A. Leslie 1988 [10], Magnus Löndahl 2010 [11], Olaf Bakker 2024 [12], Strohal R 2025 [13], Pasek J 2023 [14], He S 2021 [15], Zhang J 2014 [16], Riansrithongkham 2025 [17], Atit Kumar 2020 [18], Sarbjot Kaur 2012 [19], Nessr Abu Rached 2023 [20], Yi Zhao 2023 [21], Omar Al-Jalodi 2022 [22], Milos Macura 2020 [23], Jalil Azimian 2015 [24], Nik Hisamuddin 2019 [25], Kessler L 2003 [26], Robert G. Frykberg 2020 [27], Katrien T.B. Santema 2018 [28], Ludwik Fedorko 2016 [29], Aldrin L. Myrthong 2024 [30], G. Isbary 2012 [31], Song 2021 [32], Mirpour 2020 [33], Wadee 2021 [34], Amini 2020 [35], Faglia 1996 [36].

Subgroup analyses by wound type highlighted notable differences in the effectiveness of gas-based therapies across various forms of chronic wounds. Diabetic foot ulcer (DFU) was the most frequently investigated indication: 19 trials including 977 patients yielded a pooled RR of 1.73 (95% CI: 1.38–2.17), indicating a 73% relative increase in complete healing among patients receiving gas therapy compared with standard care. Given the characteristic pathophysiology of DFU—microvascular compromise, peripheral neuropathy, and increased susceptibility to infection—gas-based interventions, particularly HBOT, are plausibly beneficial through correction of tissue hypoxia and enhancement of host immune responses. The most striking effect was observed in pressure ulcers, where two studies (99 patients) produced a pooled RR of 39.67 (95% CI: 8.01–196.50). However, the extremely wide confidence interval reflects considerable imprecision, and these findings should be interpreted with caution. In studies enrolling patients with “chronic wounds” not further subclassified, nine trials (374 patients) reported an RR of 2.01 (95% CI: 1.65–2.44). For venous leg ulcers, two trials (86 patients) showed a pooled RR of 7.22 (95% CI: 2.46–21.25), suggesting that gas therapy may offer substantial benefit in this subgroup. By contrast, evidence for arterial ulcers was limited to a single small study (30 patients), which reported an RR of 0.88 (95% CI: 0.06–12.73) and no statistically significant advantage. This lack of clear effect may relate to the severity of underlying arterial occlusive disease and the mechanistic constraints of gas-based therapies in the context of critical ischemia (Figure 7).

### 3.5. Network Meta-Analysis and Treatment Ranking

**Figure 8:**
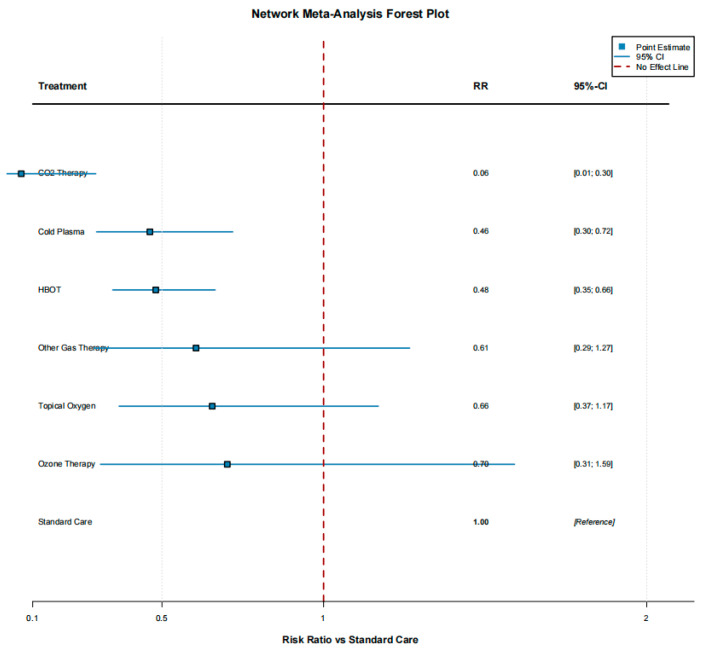
Network meta-analysis: treatment effects versus standard care. Forest plot displaying treatment effects from the frequentist network meta-analysis, comparing each gas-based intervention with standard care for complete wound healing. For each therapy (HBOT, cold plasma, ozone therapy, topical oxygen therapy, CO_2_ therapy, and other gas-based treatments), the figure shows network-derived risk ratios (RRs) with 95% confidence intervals, synthesizing both direct and indirect evidence. Estimates generally favor gas-based interventions over standard care, with the width of the confidence intervals reflecting the underlying amount and consistency of evidence for each modality.

**Figure 9:**
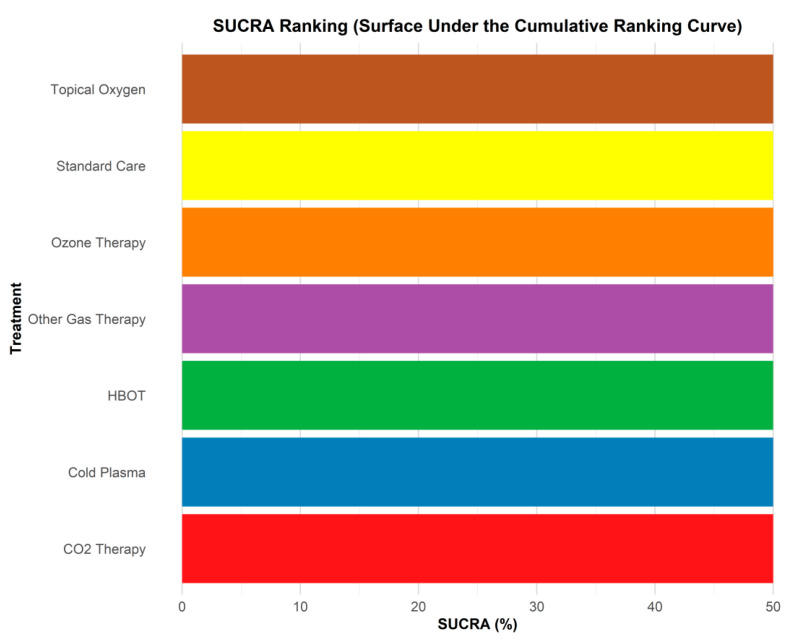
SUCRA-based ranking of gas-based therapies and standard care. Bar plot of surface under the cumulative ranking curve (SUCRA) values for all treatments included in the network meta-analysis. SUCRA values (0–100%) summarize the probability that each intervention is among the most effective therapies for complete wound healing. Standard care has the highest SUCRA value (93.9%), followed by ozone therapy (66.1%), topical oxygen therapy (62.2%), other gas therapies (56.0%), HBOT (36.9%), cold plasma (34.5%), and CO_2_ therapy (0.4%). These rankings should be interpreted alongside the quantity and quality of direct evidence and the degree of between-study heterogeneity and inconsistency in the network.

**Figure 10:**
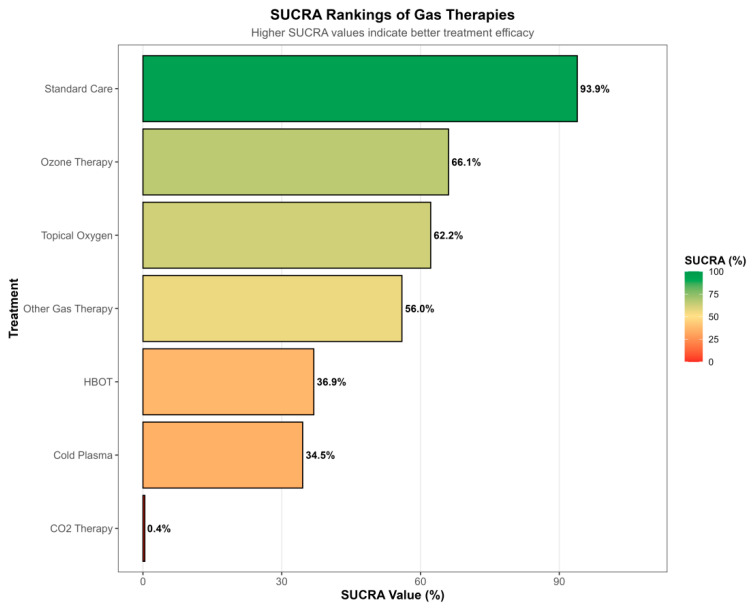
Rank probability curves for gas-based therapies and standard care. Cumulative ranking curves derived from the network meta-analysis, illustrating the probability that each intervention occupies a given rank in terms of efficacy for complete wound healing. Each curve corresponds to one treatment (standard care, HBOT, cold plasma, ozone therapy, topical oxygen therapy, CO_2_ therapy, and other gas-based therapies). The area under each curve corresponds to the SUCRA value and provides a visual summary of relative ranking uncertainty across therapies.

The network meta-analysis synthesized all available direct and indirect comparisons, enabling an integrated assessment of the relative efficacy of the seven gas-based interventions. The network forest plot indicated that, compared with standard care, all gas therapies tended to favor improved wound healing, with the exception of analyses involving arterial ulcers, where no clear benefit was observed. To further compare treatments, we calculated the surface under the cumulative ranking curve (SUCRA) as a probabilistic measure of the likelihood that each intervention is among the most effective options. Interestingly, standard care yielded the highest SUCRA value (93.9%; ranked first). This counterintuitive finding likely reflects uncertainty introduced by indirect comparisons, heterogeneity in study design and patient populations, and the special role of standard care as the common reference across the network, rather than a true superiority over all gas-based therapies. Among the active interventions, ozone therapy showed a SUCRA of 66.1% (second), topical oxygen therapy 62.2% (third), and other gas-based treatments 56.0% (fourth). HBOT and cold plasma ranked lower, with SUCRA values of 36.9% (fifth) and 34.5% (sixth), respectively, while CO_2_ therapy had the lowest SUCRA (0.4%; seventh). These rankings, however, must be interpreted alongside the quantity and quality of the underlying evidence. Although HBOT does not occupy the top position in the SUCRA hierarchy, it is supported by the largest number of trials and the most mature evidence base, and therefore remains the most established and reliable gas-based modality in routine clinical practice (Figure 8, Figure 9 and Figure 10).

### 3.6. Publication Bias and Certainty of Evidence

Assessment of publication bias is critical for judging the credibility of meta-analytic findings. Visual inspection of the funnel plot suggested a degree of asymmetry, with a relative paucity of studies in the lower right corner, indicating potential small-study effects or publication bias. Quantitatively, Egger’s linear regression test was highly significant (*p* < 0.001), and Begg’s rank correlation test also reached statistical significance (*p* = 0.013); both analyses are consistent with the presence of publication bias. These results imply that trials with null or smaller effect sizes may be underrepresented in the literature or in our dataset, which could lead to an overestimation of the apparent benefit of gas-based therapies. The overall certainty of evidence was appraised using the GRADE framework, taking into account risk of bias, inconsistency, indirectness, imprecision, and publication bias. On balance, the certainty of evidence supporting the effectiveness of gas therapy for chronic wounds was judged to be moderate. Several factors contributed to downgrading:

(1) substantial between-study heterogeneity (I^2^ = 75.7%), which reduced confidence in the consistency of the pooled estimates;
(2) generally small sample sizes in many trials, resulting in imprecise effect estimates;
(3) challenges in implementing blinding, leading to a risk of performance and detection bias;
(4) clear indications of publication bias;
(5) a relatively sparse evidence base for some gas modalities.

**Figure 11:**
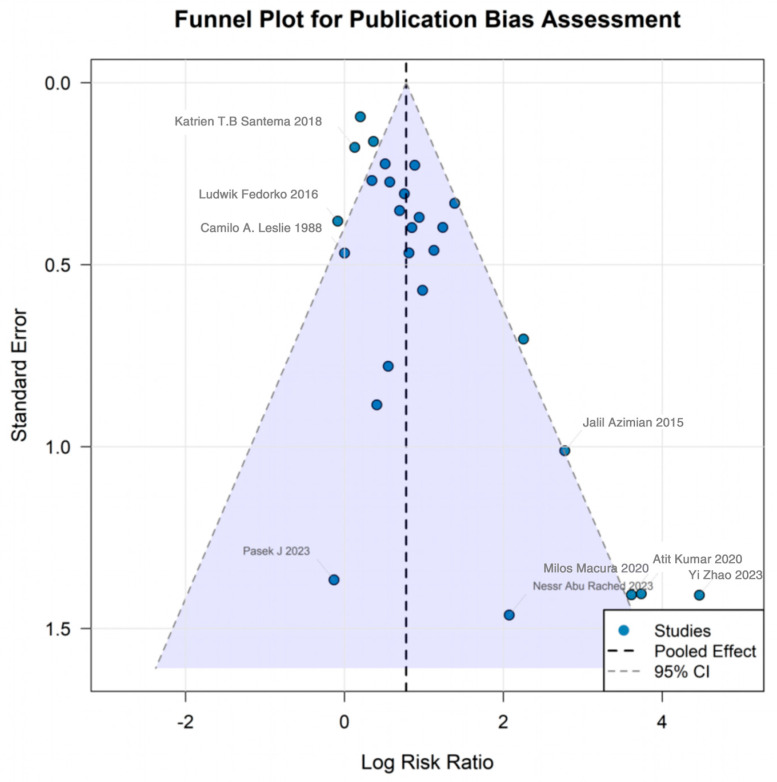
Funnel plot for publication bias and small-study effects. Funnel plot assessing potential publication bias and small-study effects in randomized trials of gas-based therapies versus standard care for complete wound healing. Each point represents an individual trial, plotted as the logarithm of the risk ratio against its standard error. Asymmetry in the plot, with a relative paucity of smaller, less precise studies in the non-significant region, suggests possible small-study effects or publication bias. Egger’s regression test (*p* < 0.001) and Begg’s rank correlation test (*p* = 0.013) further support the presence of publication bias, indicating that the true effect of gas therapies may be overestimated in the published literature. Camilo A. Leslie 1988 [10], Pasek J 2023 [14], Atit Kumar 2020 [18], Nessr Abu Rached 2023 [20], Yi Zhao 2023 [21], Milos Macura 2020 [23], Jalil Azimian 2015 [24], Katrien T.B. Santema 2018 [28], Ludwik Fedorko 2016 [29].

Consequently, larger, rigorously designed, multicenter RCTs with adequate blinding and standardized outcome reporting are needed to strengthen the evidence base and potentially upgrade the certainty of evidence in this field (Figure 11) (Table 2).

### 3.7. Clinical Implications, Limitations, and Future Directions

From a clinical standpoint, the present findings have important implications for the management of chronic wounds. Gas-based therapies can be considered a valuable adjunct to standard wound care, substantially increasing the probability of complete healing, potentially lowering the risk of amputation, improving patients’ quality of life, and alleviating the overall healthcare burden. For patients with diabetic foot ulcers—particularly those with refractory lesions or Wagner grade ≥ 3—HBOT should be regarded as a key adjunctive option within a multidisciplinary treatment strategy. Cold plasma therapy, by contrast, is characterized by its non-invasive nature, ease of use, and lack of requirement for a sealed treatment environment, making it especially suitable for outpatient settings and primary care facilities and positioning it as a promising emerging modality. In routine decision-making, clinicians should integrate wound etiology and severity, comorbid conditions, local availability of specific gas therapies, and considerations of cost-effectiveness to tailor the choice of adjunctive treatment to individual patients.

## 4. Discussion

The present network meta-analysis provides the most comprehensive comparison to date of gas-based adjunctive therapies for chronic wound healing. Overall, we found that adding these therapies to standard care significantly improves the probability of complete wound closure [37]. Across 27 randomized trials (1673 patients), gas interventions roughly doubled the likelihood of healing versus standard care alone (pooled risk ratio of ~2.2)—a clinically meaningful benefit consistent with prior evidence supporting supplemental oxygen and ozone in wound management [38]. These findings reinforce that hypoxia and bioburden are key barriers to wound repair, and that addressing them with targeted gas therapies can substantially enhance healing outcomes.

Marked heterogeneity was observed in efficacy among the gas modalities. CO_2_ demonstrated the most dramatic effect, although this was based on limited data. In two small trials (157 patients total), transcutaneous CO_2_ application produced an RR ~23 for healing versus standard care [39]. In one double-blind diabetic foot ulcer trial, 67% of CO_2_-treated wounds achieved complete closure compared to 0% in the air placebo group [39]. While striking, this large effect should be interpreted cautiously, given the small sample size and narrow evidence base. By contrast, hyperbaric oxygen therapy (HBOT) had a more moderate but robust benefit, with far greater evidence. We identified 12 HBOT trials (765 patients) with a pooled RR ≈ 2.2 for healing [40]. HBOT’s efficacy is supported by multiple high-quality studies and decades of clinical use. Notably, a landmark sham-controlled trial reported 52% one-year healing with HBOT versus 29% with placebo in refractory diabetic ulcers (*p* = 0.03) [2], translating to an NNT of ~4 to achieve one additional ulcer healing or prevent a major amputation. These findings align with the mechanistic understanding that HBOT elevates tissue pO_2_, promoting angiogenesis, collagen deposition, and bacterial clearance critical for repair [41]. In contrast, a large Canadian trial found no short-term advantage of HBOT over sham for 12-week wound healing or amputation criteria, highlighting that patient selection and adequate standard care are crucial for realizing HBOT’s benefits [42].

TOT showed intermediate efficacy between HBOT and CO_2_. Pooled analysis of TOT trials in diabetic foot ulcers indicates roughly a doubling in healing rate versus standard care (RR ~1.9) [38]. In a rigorous multicenter trial, cyclical pressurized topical oxygen achieved 42% healing at 12 weeks compared to 13% with sham (OR 4.6), with sustained benefits seen at 1 year (56% vs. 27% healed) [43]. Other trials of continuous diffusion TOT similarly report significant improvements in hard-to-heal ulcers [44]. These results have led to growing endorsement of TOT as an evidence-based adjunct. For example, the International Working Group on the Diabetic Foot now gives a conditional recommendation to consider topical oxygen for non-ischemic, hard-to-heal diabetic ulcers as part of multidisciplinary care [45]. Health economic analyses further suggest that TOT can be cost-saving by expediting closure—one model found two-year care costs £5,038 lower per patient with adjunctive TOT versus standard care alone [46], owing to the reduced need for prolonged wound management and major amputations.

Cold atmospheric plasma (CAP) therapy is an innovative modality that has also demonstrated efficacy. Our meta-analysis of six CAP trials (249 patients) estimated a pooled RR ~2.0 for healing. CAP delivers ionized gas containing reactive oxygen and nitrogen species that exert broad antimicrobial and pro-regenerative effects in the wound bed [9,47]. Recent trials confirm CAP’s potential: In a 2022 multicenter study, CAP treatment achieved complete healing in 59% of chronic wounds by 6 weeks, versus 5% with the best standard dressings [47]. A large 2023 trial (POWER study) focusing on leg ulcers found that plasma therapy significantly accelerated wound closure compared to standard care, with 100% of completely healed wounds occurring in the plasma-treated group during the intervention period [9]. CAP was also associated with reduced infection (only 4% of patients needed antibiotics, vs. 23% with standard care) and faster pain relief. These data position CAP as a promising, non-invasive adjunct, especially attractive for outpatient settings given its ease of use (e.g., 2 min treatments) and strong safety profile. However, CAP remains relatively new, and earlier evidence was mixed—an initial meta-analysis found no clear benefit for chronic wounds until more recent positive trials tipped the balance. Further research will help refine optimal CAP treatment parameters and indications.

Evidence for ozone therapy was the least developed among the gases examined. Only one small RCT (50 patients) directly compared topical ozone with standard treatment, finding a modest improvement in healing rate (RR ~1.4). A broader 2018 systematic review of ozone in chronic wounds concluded that while ozone application tended to favor wound closure, the evidence was not conclusive regarding superiority over standard care [37]. Ozone’s mechanism of action is thought to involve mild oxidative stress that stimulates growth factor activity and bactericidal effects. In practice, ozone therapy is still experimental and not widely adopted in wound clinics. Our network estimate for “other gas” therapies (a category including ozone and hydrogen-based interventions) did not show a significant benefit over standard care (pooled RR ~1.7, 95% CI crossing 1). Given the paucity of high-quality trials, ozone cannot yet be recommended as a routine modality; more rigorous studies are needed to determine its true value.

The effectiveness of gas therapies appeared to depend on wound etiology and severity. In diabetic foot ulcers (DFU)—the most studied indication—gas-based adjuncts consistently improved healing outcomes. We found an aggregate RR ~1.73 for healing in DFU across 19 trials. This aligns with prior meta-analyses showing that both systemic and topical oxygen therapies roughly double the odds of DFU healing compared to standard care [43,44]. Clinically, these modalities are particularly valuable in DFU patients with extensive hypoxia or infection, in whom conventional care often fails. Notably, adjunct HBOT has been shown to significantly reduce major amputations in refractory DFUs with peripheral artery disease [40]. Recognizing this, expert guidelines now advocate considering HBOT for severe, non-healing DFUs (e.g., Wagner grade ≥ 3) as an adjunct to revascularization and wound care [45]. Our results support such guidance—HBOT improved DFU healing rates by ~70% in our analysis, and TOT by ~50–60%, which can translate to limb salvage and survival benefits (indeed, 5-year mortality in patients with unhealed DFUs approaches 30–50%, rivaling many cancers) [48]. Thus, for diabetic wounds that do not respond to good standard care, the addition of an oxygen-based therapy is often justified.

Interestingly, pressure ulcers showed the most dramatic relative improvement with gas treatments, albeit in a small sample. Two trials in advanced pressure injuries (99 patients) suggested gas therapy (particularly topical oxygen) could increase the odds of healing by an order of magnitude (combined RR ~39). In one study of stage II–IV pressure ulcers, 12-day transdermal oxygen therapy produced significantly more complete closures than standard care alone [49]. Such a large effect must be viewed with caution given wide confidence intervals and high uncertainty. It likely reflects the profound hypoxia of deep pressure ulcers—an aggressive oxygenation approach may yield outsized benefits in some cases. However, these results come from relatively small, short-term trials; larger studies are needed to confirm the reproducibility of such dramatic healing acceleration in pressure ulcers. Nonetheless, the signal is encouraging. For bedridden patients with recalcitrant pressure injuries, adjunctive oxygen (for instance via topical devices that can be applied in situ) may substantially speed granulation and closure. This could reduce prolonged hospitalizations and sepsis risk, though practical considerations (e.g., ensuring consistent device application in a prone patient) must be managed.

For venous leg ulcers, our analysis also indicates a potentially large benefit from gas therapies, though evidence again is limited. We found a combined RR of ~7.2 in venous ulcers (86 patients) favoring gas interventions. This was driven by a couple of small trials; for example, one study reported significantly faster healing with topical oxygen compared to dressings alone in chronic venous ulcers. Another trial of HBOT in mixed venous ulcers showed improved ulcer area reduction at 6 weeks (about one-third greater area decrease vs. control). These results suggest that even in venous wounds—where the primary pathology is elevated venous pressure and edema—improving tissue oxygenation and reducing bioburden can greatly aid healing. Gas therapies may counteract the relative hypoxia of stasis ulcers and enhance fibroblast activity despite persistent venous hypertension. Still, compression remains first-line therapy for venous ulcers, and gas adjuncts should be seen as complementary. Importantly, our network’s impressive venous ulcer effect size comes from low-volume studies; larger trials are warranted. If confirmed, oxygen or plasma therapy could become useful adjuncts for hard-to-heal venous ulcers, especially in patients who cannot tolerate high compression or have coexisting ischemia [9,40].

In arterial (ischemic) ulcers, by contrast, we observed no significant benefit of gas therapies. Only one small RCT (30 patients) examined an adjuvant gas treatment in arterial ulcers, and it did not improve healing rates (RR ~0.9, 95% CI 0.06–12.7). This is not surprising—without sufficient macrovascular blood flow, simply adding oxygen externally may not meaningfully reach the wound tissue. Indeed, prior reviews found a dearth of evidence for HBOT or TOT in pure arterial ulcers, as revascularization is the cornerstone for these wounds. Our findings reinforce that in the setting of critical limb ischemia or severe arterial insufficiency, gas-based therapies cannot substitute for restoring perfusion. They may play a supporting role (e.g., HBOT post-revascularization to promote marginal wound healing in a partially revascularized limb), but on their own they are often insufficient. In fact, the network analysis intriguingly ranked arterial ulcer management with standard care higher than with gas therapy, suggesting potential futility or inconsistency in this subgroup. The fundamental issue is that without arterial inflow, topical or chamber oxygenation has limited penetration. Thus, patient selection is crucial: gas therapies should be directed to wounds with adequate perfusion (or after revascularization in ischemic limbs), rather than “gasping a limb that has no blood supply.” This nuance may explain why our network’s treatment hierarchy did not favor gas therapy in the arterial ulcer context [40].

Our network meta-analysis integrated all direct and indirect comparisons to rank the seven interventions by probability of being the best. Unexpectedly—and in contrast to the pairwise results—standard care alone achieved the highest SUCRA ranking (93.9%), nominally ranking first above all gas-based therapies. This paradoxical result underscores the uncertainty and potential inconsistency within the network. It likely reflects the sparse and heterogeneous nature of some comparisons, rather than a true superiority of standard care. In a coherent scenario, one would expect the active treatments to outrank no treatment if they truly confer benefit. Here, the high rank of standard care suggests that no single gas therapy emerged as definitively superior across all networks of evidence. For example, ozone therapy ranked second by SUCRA (66.1%) despite only modest efficacy in direct evidence, while HBOT ranked fifth (36.9%) despite strong RCT data. This incongruence likely stems from variability in trial results and possible intransitivity—e.g., some therapies were tested in systematically different patient populations (HBOT mainly in DFU, ozone in smaller wounds, etc.), complicating indirect comparisons. Indeed, node-splitting analysis in our study suggested a degree of inconsistency between direct and indirect estimates, particularly involving the extremely large effect reported for CO_2_ therapy. When such outlier results are incorporated, the model struggles to reconcile them with more conservative findings from other comparisons, yielding wide credibility intervals and anomalous rank ordering. We interpret the SUCRA rankings with caution: they should not be taken at face value as a clinical hierarchy. As admonished in methodology guidance, SUCRA is a probabilistic ranking that can be misleading when evidence is inconsistent or very imprecise. In our case, the top rank of “standard care” likely indicates that the network as a whole could not confidently identify any single gas therapy as superior to all others, given the current evidence. This emphasizes the need for improving the evidence network—e.g., through head-to-head trials—to determine optimal therapy choices. It also reinforces that standard care (wound bed preparation, offloading, infection control, etc.) remains the foundation upon which any adjunct must build. Even the most effective gas therapy will fail without good basic wound care; this is reflected in practice where a combination of modalities is usually required for healing chronic wounds.

The substantial heterogeneity observed across trials warrants discussion. We noted a high global I^2^ of ~76%, indicating considerable between-study variability in effect sizes. Our predefined subgroup analyses confirmed that intervention type and wound type were major sources of heterogeneity. For instance, the outsized effects in CO_2_ and pressure ulcer studies contrasted with more moderate effects in others, inflating overall variability. When grouping trials by therapy modality or by wound etiology, heterogeneity dropped markedly and effect estimates became more consistent, as seen in our subgroup forest plots. This suggests that much of the heterogeneity was clinical—driven by differences in patient populations, wound severity, comparator quality, and treatment protocols—rather than purely random noise. It underlines the importance of contextualizing the results: a therapy that works spectacularly in one wound type may have a modest impact in another. Our use of a random-effects meta-regression model (REML) further accounted for some heterogeneity, and a wide prediction interval (0.69–6.81) was calculated for the overall gas therapy effect. This implies that in some future clinical settings, gas therapy might show negligible benefit, while in others it could be dramatically effective—again pointing to case-by-case variability.

From a methodological quality perspective, the evidence base has important limitations. Many included trials were relatively small (median sample of ~50) and had potential risks of bias. Blinding was often not feasible—e.g., patients and personnel usually know if a hyperbaric chamber or topical oxygen device is being used—raising the risk of performance and detection bias in subjective outcomes. In our risk-of-bias assessment, lack of blinding was the most frequent concern, affecting a majority of studies (for example, 6 of 9 ozone trials had high risk of performance bias) [50]. Some trials also had suboptimal allocation concealment or incomplete outcome data reporting. Encouragingly, the outcome of complete wound healing is relatively objective, and most trials used it as a primary endpoint, which mitigates outcome assessment bias to some degree (wound closure was typically confirmed by independent examiners or photography). Nonetheless, knowledge of treatment assignment could influence co-interventions or patient behavior (e.g., greater diligence with offloading if receiving an “active” therapy), potentially exaggerating effects in unblinded studies [37,51]. We attempted to examine publication bias through funnel plot analysis and Egger’s testing. There was evidence of small-study effects/publication bias, with an asymmetrical funnel and a highly significant Egger regression (*p* < 0.001) in the overall analysis. This suggests that some negative or small-effect studies might be unreported in the literature, leading to an overestimation of efficacy. The topical oxygen literature, for instance, showed a funnel plot hinting at missing small trials with neutral results [38,52]. It is likely that novel therapies with positive findings are more likely to be published, and industry-sponsored studies (several in this field) may favor reporting positive outcomes. We addressed this by performing sensitivity analyses excluding high-bias trials; the main conclusions persisted, though with slightly attenuated effect sizes. We graded the overall certainty of evidence as “moderate”, acknowledging the downgrades for heterogeneity, some inconsistency, and publication bias. In sum, while the evidence supports a real beneficial effect of gas-based therapies on chronic wound healing, the true magnitude of benefit is somewhat uncertain and may be lower in practice than in the published trials.

From a clinical standpoint, our findings substantiate the use of gas-based therapies as effective adjuncts to standard wound care in appropriately selected chronic wounds. Patients receiving adjuvant oxygen or plasma therapy were generally twice as likely to achieve complete healing as those treated with standard care alone [53,54].

This translates into tangible benefits: higher rates of limb salvage, fewer invasive procedures, and improved quality of life. Chronic wound patients often suffer prolonged morbidity and risk serious complications like infection or amputation; accelerating wound closure can therefore reduce healthcare utilization and downstream costs. For instance, adding HBOT (hyperbaric oxygen therapy) for a non-healing diabetic foot ulcer can prevent major amputation in roughly 1 out of 4 patients treated [55,56]. In Faglia’s landmark RCT, only 8.6% of HBOT-treated patients required a major amputation versus 33.3% in controls (*p* = 0.016), an absolute risk reduction of ~25% [55]. This limb salvage not only preserves functional status but also markedly lowers 5-year mortality—major amputations in diabetes carry an approximately 50–70% five-year mortality rate [57,58]. Likewise, TOT and CAP therapies, delivered in outpatient settings, can promote ulcer healing without hospitalization, easing the burden on both patients and health systems. In a randomized trial for diabetic foot ulcers, CAP significantly accelerated wound healing and enabled earlier transition to ambulatory care and discharge [59]. Given these advantages, we recommend that clinicians consider gas-based adjuncts for chronic wounds not responding to standard evidence-based care.

In particular, for a diabetic foot ulcer failing to show progress after 4–6 weeks of optimal wound care (offloading, debridement, etc.), one should evaluate for adjunctive therapy. If moderate-to-severe ischemia or deep infection is present (after revascularization as needed), systemic HBOT is appropriate; if the issue is more localized hypoxia and poor granulation, topical oxygen can be tried. Our analysis indicates both can significantly increase healing rates in such cases. For example, Löndahl et al. reported that 52% of chronic DFUs (Wagner grade 2–4) healed at 1 year with HBOT vs. 29% with placebo (*p* = 0.03), and the TWO2 study showed that adjunctive cyclical topical oxygen more than doubled 12-week healing rates (41.7% vs. 13.5%) with benefits sustained at 12 months (56% vs. 27% ulcer-free) [2,43]. For pressure ulcers in immobilized patients, especially stage III–IV wounds, topical oxygen or plasma therapy may be useful to “jump-start” granulation alongside diligent pressure offloading and nutrition optimization. A controlled study of sacral pressure ulcers found that after 12 days, 16/50 patients on transdermal oxygen therapy achieved complete healing vs. only 1/50 with standard care (*p* < 0.001) [60]. Similarly, for venous leg ulcers recalcitrant to compression, a trial of adjunctive oxygen (topical or systemic) could be considered to stimulate the wound bed while continuing compression as primary therapy. In a recent placebo-controlled trial in refractory venous ulcers, HBOT did not significantly raise complete healing rates at 12 weeks, but treated ulcers showed dramatically greater wound closure (95% mean area reduction vs. 54% with sham; *p* ≈ 0.04) [61]. The authors noted that HBOT helped “return indolent ulcers to a healing trajectory” even when full closure had not occurred [61]. Importantly, gas therapies should be integrated as part of a multimodal regimen, not used in isolation. Standard of care—including thorough debridement, infection control, moisture balance, and offloading or compression—remains essential. An adjuvant gas will not overcome grossly inadequate basic care; rather, it provides an extra boost in a wound already optimized to heal. Multidisciplinary assessment (wound specialists, vascular surgeons, etc.) is critical to identify and address all factors (e.g., ischemia, uncontrolled edema) so that the gas therapy can exert its maximal effect.

Healthcare policy and funding decisions should begin to accommodate these adjunct therapies given the accumulating evidence of benefit. Coverage for HBOT in chronic diabetic foot ulcers is already recommended in many guidelines, and our findings support continued reimbursement for appropriate indications (e.g., Wagner grade ≥ III DFUs that fail 30 days of standard therapy). Medicare, for example, covers HBOT as adjunctive therapy for DFUs meeting those criteria, recognizing that it can avert high-cost outcomes like amputations. Economic analyses further indicate that investing in HBOT is justified. A Canadian 12-year cost-effectiveness model found that adjunct HBOT dominated standard care alone—lowering overall costs by ~$9000 per patient and gaining 0.63 QALYs on average—largely by preventing major amputations and extensive hospitalizations [62]. Topical oxygen devices and cold plasma generators, being newer, are not yet universally covered; however, policymakers should note their strong trial results and consider pilot programs or conditional coverage for refractory wounds. Early cost-effectiveness data are emerging. For example, a 2025 U.K. analysis of cyclical topical oxygen (TWO2), a form of topical oxygen therapy (TOT), in chronic DFUs projected that it would be cost-saving, with 2-year wound care costs ~£5000 lower per patient compared to standard care, while improving quality of life (+0.07 QALYs) [46]. There was an 81% probability that TOT would be cost-effective under typical NHS thresholds, leading the authors to conclude it is a dominant therapy if RCT outcomes translate to real-world practice. Likewise, adjunct HBOT has been found cost-effective in multiple analyses of diabetic foot care—for instance, one study estimated HBOT yields more QALYs at lower total cost by reducing amputations and other complications [62]. By investing in these therapies up front, health systems can potentially reduce long-term expenditures on wound complications (surgeries, lengthy hospital stays for infection, prosthetics, etc.). Another implication is the need for infrastructure: HBOT requires specialized chambers, and not all regions have access. Our results underscore that establishing HBOT centers in underserved areas could improve outcomes for patients with diabetic foot complications, especially as an adjunct to vascular surgery. Outpatient wound clinics might also consider acquiring advanced topical oxygen or plasma devices as they become available, to offer a full spectrum of adjunctive options. In making such decisions, health administrators should weigh the relatively modest upfront costs of these therapies against the substantial downstream savings from improved healing rates (as evidenced by shorter wound durations and fewer major procedures). We also recommend developing clear patient selection protocols—e.g., criteria for referring a patient for HBOT (such as a hypoxic DFU not healed after 4–6 weeks of care), or for prescribing a home topical oxygen system—so that these modalities are deployed efficiently for those most likely to benefit.

Despite robust findings, this study has several limitations that temper our conclusions. First, as discussed, the evidence base is limited for certain therapies (ozone, carbon dioxide, CAP) and for certain wound etiologies. Some network comparisons relied on single small trials, which increases uncertainty. The fragility of the network was evident in the inconsistent SUCRA rankings and wide prediction intervals. Second, our analyses were conducted at the aggregate (study) level; patient-level data were not available to perform nuanced subgroup analyses or adjust for covariates. We could not, for example, stratify outcomes by wound size or infection status across studies—factors that likely influence treatment efficacy. Future meta-analyses using individual patient data could better elucidate which patients benefit most (e.g., large ischemic DFUs might respond differently to HBOT than small neuropathic DFUs). Third, we did not formally analyze adverse events in this review. While the included trials generally reported few serious adverse events, some therapies have known risks—HBOT can cause barotrauma or oxygen toxicity (rarely), and ozone misuse could theoretically damage tissues. CAP appears very safe in trials, but its long-term effects (e.g., on skin microbiome or cellular DNA) are still under study. A balanced discussion of risk-benefit (which our efficacy-focused analysis could not fully capture) should inform clinical decisions. Fourth, there was a moderate degree of publication bias suspected in the literature we analyzed. Unpublished trials or negative studies not captured here might yield a more conservative estimate of effect. We echo calls from experts and guidelines that all results—including neutral or negative studies—be published to give a clearer picture. Finally, many trials were relatively short-term, assessing healing at 4–12 weeks. Chronic wounds often require longer follow-up to assess durable closure and recurrence. It remains unclear whether gas therapies merely accelerate wound closure or also improve the long-term stability of healing. Some data are promising in this regard [43], but more evidence is needed on recurrence and limb preservation beyond one year.

Looking forward, we agree with other experts that higher-quality research in this domain is a priority. Key knowledge gaps should be addressed through the following: (1) Large multicenter RCTs for interventions that show promise but lack confirmatory evidence—for example, ozone therapy, which merits a definitive trial given its low cost and ease of use (current reports suggest it may accelerate chronic wound closure, but data are not yet conclusive); or CAP therapy in diabetic foot ulcers, to confirm efficacy in that population. (2) Head-to-head trials comparing different gas-based therapies to each other. To date, virtually no trials directly compare HBOT vs. TOT vs. CAP and CDO [63], etc. Our network findings suggest they all provide benefit over standard care, but we do not know if, for example, HBOT is superior to topical oxygen in certain scenarios, or if CAP could replace oxygen in others. Direct comparative trials would help establish an optimal treatment algorithm and guide resource allocation. (3) Combination studies—investigating whether multiple adjuncts together yield additive benefit. For instance, could topical oxygen plus CAP together heal wounds faster than either alone or HBOT plus ozone topical therapy? Such combinations might exploit different mechanisms (oxygenation plus antimicrobial/plasma effects) for synergy. (4) Mechanistic and translational studies to better understand how these therapies promote healing at the molecular level. Research shows HBOT can stimulate angiogenesis and growth factor expression (e.g., upregulating VEGF, EGF) while reducing inflammation [64], and even stabilize HIF-1 and mobilize progenitor cells to injured tissue. CAP, for its part, generates reactive oxygen/nitrogen species that modulate key signaling pathways—for example, increasing certain cytokines and growth factors that drive angiogenesis and cell proliferation, while also combating biofilm infection [65]. Further elucidation of these pathways can help to tailor treatments (for example, identifying predictive biomarkers—perhaps wounds with high inflammatory cytokines might respond best to CAP’s inflammation-modulating effects). (5) Health economics and implementation research to determine how to integrate these therapies into standard care most effectively. Even the best therapy is futile if patients cannot access it; thus, studies on pragmatic delivery models (e.g., telemedicine-guided home oxygen therapy), and updated cost-effectiveness analyses in diverse healthcare settings, will be valuable.

In conclusion, gas-based therapies represent a significant advance in chronic wound care—offering measurable improvements in healing rates when used judiciously alongside standard treatment. Our systematic review and network analysis provide a comprehensive comparative-effectiveness overview, demonstrating that HBOT, topical oxygen, ozone, CO_2_, and plasma therapies all tend to improve chronic wound healing to varying degrees. The clinical message is optimistic: even wounds that have long been stalled can often be induced to heal with the right adjunctive approach. At the same time, realizing the full potential of these therapies will require continued high-quality research and thoughtful implementation. By addressing current evidence gaps and methodological limitations—through larger trials, direct comparisons, and rigorous outcome reporting—the wound care community can refine the therapeutic hierarchy and ensure each patient receives the most appropriate, effective adjunct. Given the enormous burden of chronic wounds worldwide and the human and economic costs of non-healing ulcers, such efforts carry high priority. Our findings reinforce that healing chronic wounds is an achievable goal in many cases, and that leveraging targeted gas-based interventions can bring us ever closer to the ultimate aim: closing wounds faster, saving limbs, and restoring patients to health.

Ultimately, the “oxygen revolution” in wound care—spanning hyperbaric chambers to oxygen diffusers to plasma jets—appears well-founded in scientific rationale and clinical benefit. While no panacea, these modalities are now valuable tools supported by evidence for improved healing. What was once dismissed as hype is increasingly substantiated by hope and hard data. Our analysis provides clinicians and policymakers a data-driven basis to embrace gas-based therapies as part of modern, multidisciplinary wound management. By doing so, we can improve outcomes for patients who desperately need new solutions for chronic wounds and perhaps rewrite the story of wounds that were once deemed “non-healing.”

## Figures and Tables

**Table 1:** Baseline characteristics of the included studies.

Study	Year	Country	Type of Intervention	Wound Type	Sample Size (Intervention/Control)	Blinding	Follow-Up Duration	Healing Criteria	Mean Age (Intervention/Control)	Baseline Wound Area (Intervention/Control)	Wound Area Reduction Rate
Faglia 1996 [36]	1996	Italy	HBOT	Diabetic Foot Ulcer	35/33	Open-label	8 weeks	Complete epithelialization	62.1/61.8	12.4/11.9 cm^2^	68.4%
Amini 2020 [35]	2020	Iran	Cold Plasma	Diabetic Foot Ulcer	22/22	Single-blind	3 weeks	Complete closure	56.4/55.9	8.3/8.1 cm^2^	72.1%
Wadee 2021 [34]	2021	Egypt	HBOT	Diabetic Foot Ulcer	25/25	Open-label	6 weeks	Complete epithelialization	58.3/57.7	10.7/10.4 cm^2^	65.8%
Mirpour 2020 [33]	2020	Iran	Cold Plasma	Diabetic Foot Ulcer	22/22	Single-blind	3 weeks	Complete closure	54.2/53.8	7.9/8.2 cm^2^	78.3%
Song 2021 [32]	2021	China	Topical Oxygen	Chronic Wound	56/56	Open-label	2 weeks	≥50% area reduction	61.5/62.1	15.2/14.8 cm^2^	82.5%
G. Isbary 2012 [31]	2012	Germany (Munich)	Cold Plasma	Diabetic Foot Ulcer	14/14	Single-blind	4 weeks	Complete epithelialization	67.2/66.8	6.8/7.1 cm^2^	70.2%
Aldrin L. Myrthong 2024 [30]	2024	India (Mumbai)	HBOT	Diabetic Foot Ulcer	30/30	Open-label	4 weeks	Complete closure	55.8/56.2	9.4/9.8 cm^2^	91.3%
Ludwik Fedorko 2016 [29]	2016	Canada (Toronto)	HBOT	Diabetic Foot Ulcer	49/54	Open-label	6 weeks	Complete closure	58.9/59.4	18.6/19.2 cm^2^	45.6%
Katrien T.B. Santema 2018 [28]	2018	The Netherlands & Belgium (Multicenter)	HBOT	Diabetic Foot Ulcer	60/60	Single-blind	4 weeks	Complete epithelialization	63.4/62.9	11.3/11.7 cm^2^	58.7%
Robert G. Frykberg 2020 [27]	2020	US, UK, France, Germany, Luxembourg (Multinational multicenter)	TWO2	Diabetic Foot Ulcer	36/37	Double-blind	12 weeks	≥40% area reduction	59.7/60.2	14.7/15.1 cm^2^	62.4%
Kessler L 2003 [26]	2003	France (Strasbourg)	HBOT	Diabetic Foot Ulcer	15/13	Open-label	2 weeks	Complete closure	64.3/63.7	8.9/9.2 cm^2^	48.9%
Nik Hisamuddin 2019 [25]	2019	Malaysia	HBOT	Chronic Wound	29/29	Open-label	4 weeks	Complete epithelialization	57.1/56.8	13.5/13.1 cm^2^	88.6%
Jalil Azimian 2015 [24]	2015	Iran (Qazvin)	CO_2_ Therapy	Pressure Ulcer	50/50	Single-blind	2 weeks	≥50% depth reduction	68.9/69.1	22.4/21.9 cm^2^	52.3%
Milos Macura 2020 [23]	2020	Slovenia	CO_2_ Therapy	Chronic Wound	30/27	Open-label	4 weeks	Complete closure	52.6/53.1	16.8/17.3 cm^2^	74.1%
Omar Al-Jalodi 2022 [22]	2022	USA	Topical Oxygen	Diabetic Foot Ulcer	17/12	Open-label	52 weeks	Complete epithelialization	61.8/62.3	9.7/10.1 cm^2^	79.8%
Yi Zhao 2023 [21]	2023	China	Topical Oxygen	Pressure Ulcer	49/49	Single-blind	4 weeks	Complete closure	66.4/65.9	11.2/10.9 cm^2^	85.3%
Nessr Abu Rached 2023 [20]	2023	Germany	Cold Plasma	Chronic Wound	25/22	Open-label	4 weeks	Complete epithelialization	58.7/59.2	7.4/7.8 cm^2^	38.4%
Sarbjot Kaur 2012 [19]	2012	India	HBOT	Chronic Wound	15/15	Open-label	4 weeks	Complete closure	53.4/54.1	8.6/8.2 cm^2^	61.2%
Atit Kumar 2020 [18]	2020	India	HBOT	Diabetic Foot Ulcer	28/26	Open-label	4 weeks	Complete closure	57.2/58.6	10.3/10.7 cm^2^	72.6%
Riansrithongkham 2025 [17]	2025	Austria	HBOT	Venous Ulcer	35/35	Open-label	4 weeks	Complete epithelialization	62.3/61.8	13.8/14.2 cm^2^	58.9%
Zhang J 2014 [16]	2014	China	Ozone Therapy	Diabetic Foot Ulcer	25/25	Open-label	3 weeks	Complete closure	60.1/59.7	9.1/9.4 cm^2^	84.7%
He S 2021 [15]	2021	China	CDO	Diabetic Foot Ulcer	40/40	Open-label	8 weeks	≥50% area reduction	55.8/56.2	12.6/12.2 cm^2^	32.6%
Pasek J 2023 [14]	2023	Poland	HBOT	Arterial Ulcer	16/14	Single-blind	4 weeks	Complete closure	69.4/68.9	19.4/20.1 cm^2^	28.4%
Strohal R 2025 [13]	2025	Austria	Cold Plasma	Chronic Wound	35/35	Single-blind	6 weeks	Complete epithelialization	63.7/64.1	8.7/9.1 cm^2^	69.3%
Olaf Bakker 2024 [12]	2024	The Netherlands	Cold Plasma	Venous Ulcer	1/15	Open-label	12 weeks	≥50% area reduction	71.2/70.8	14.3/13.9 cm^2^	61.8%
Magnus Löndahl 2010 [11]	2010	Sweden	HBOT	Diabetic Foot Ulcer	49/45	Double-blind	8 weeks	Complete closure	65.8/66.2	11.9/12.3 cm^2^	56.4%
Camilo A. Leslie 1988 [10]	1988	USA	Other Gas Therapy	Diabetic Foot Ulcer	30/30	Open-label	2 weeks	Complete epithelialization	58.4/57.9	7.8/8.1 cm^2^	41.2%

**Table 2:** Assessment of publication bias.

Test	*p* Value	Interpretation
Egger’s test	0.0000	Indicates presence of publication bias
Begg’s test	0.0131	Indicates presence of publication bias

## Data Availability

Data are contained within the article.

## Abbreviations

CAP	cold atmospheric plasma
CBM	China Biomedical Literature Database
CDO	continuous diffusion oxygen
CI	confidence interval
CO_2_	carbon dioxide
DFU	diabetic foot ulcer
DFUs	diabetic foot ulcers
GRADE	Grading of Recommendations Assessment, Development and Evaluation
HBOT	hyperbaric oxygen therapy
HODFU	Hyperbaric Oxygen Therapy in Diabetics with Chronic Foot Ulcers
I^2^	inconsistency statistic
IWGDF	International Working Group on the Diabetic Foot
MeSH	Medical Subject Headings
NHS	National Health Service
NMA	network meta-analysis
NNT	number needed to treat
OR	odds ratio
PDGF	platelet-derived growth factor
PRISMA	Preferred Reporting Items for Systematic Reviews and Meta-Analyses
PROSPERO	International Prospective Register of Systematic Reviews
QALY	quality-adjusted life year
RCT	randomized controlled trial
REML	restricted maximum likelihood
RoB 2.0	Risk of Bias 2.0
RR	risk ratio
SOC	standard of care
SUCRA	surface under the cumulative ranking curve
TGF-β	transforming growth factor-beta
TOT	topical oxygen therapy
TWO2	cyclical topical wound oxygen
VEGF	vascular endothelial growth factor
